# Cloning and Expression of TNF Related Apoptosis Inducing Ligand in *Nicotiana tabacum*

**Published:** 2015

**Authors:** Hamid Reza Heidari, Mojgan Bandehpour, Hossein Vahidi, Jaleh Barar, Bahram Kazemi, Hossein Naderi-Manesh

**Affiliations:** a *Department of Pharmaceutical Biotechnology, Faculty of Pharmacy, Shahid Beheshti University of Medical Sciences, Tehran, Iran.*; b *Cellular and Molecular Biology Research Center, Shahid Beheshti University of Medical Sciences, Tehran, Iran.*; c *Department of Biotechnology, School of Medicine, Shahid Beheshti University of Medical Sciences, Tehran, Iran.*; d *Research Centre for Pharmaceutical Nanotechnology, Tabriz University of Medical Sciences, Tabriz, Iran.*; e *Ovarian Cancer Research Center, University of Pennsylvania, Philadelphia, USA.*; f *Department of Biophysics, Faculty of Biological Sciences, Tarbiat Modares University, Tehran, Iran.*

**Keywords:** Recombinant protein, TRAIL, Molecular Farming, Nicotiana tabacum, *Agrobacterium tumefaciens*

## Abstract

Molecular farming has been considered as a secure and economical approach for production of biopharmaceuticals. Human TNF Related Apoptosis Inducing Ligand (TRAIL) as a promising biopharmaceutical candidate has been produced in different expression hosts. However, little attention has been paid to molecular farming of the TRAIL in spite of numerous advantages of plant expression systems. Therefore, in this study the cytoplasmic production of the TRAIL was tackled in *Nicotiana tabacum* using *Agrobacterium tumefaciens *LBA 4404*.* Initially, the desired coding sequence was obtained using PCR technique on the constructed human cDNA library. Afterward, the necessary requirements for expression of the TRAIL in plant cell system were provided through sub-cloning into 35S-CaMV (Cauliflower Mosaic Virus) helper and final 0179-pGreen expression vectors. Then, the final TRAIL-pGreen expression vector was cloned into *A. tumefaciens *LBA 4404*.* Subsequently, the *N. tabacum* cells were transformed through co-culture method and expression of the TRAIL was confirmed by western blot analysis. Finally, the recombinant TRAIL was extracted through chromatographic technique and biological activity was evaluated through MTT assay (Methylthiazol Tetrazolium Assay). The result of western blot analysis indicated that only monomer and oxidized dimer forms of the TRAIL can be extracted from the *N. tabacum* cells. Moreover, the lack of trimeric assembly of the extracted TRAIL diminished its biological activity in sensitive A549 cell line. In conclusion, although *N. tabacum* cells can successfully produce the TRAIL, proper assembly and functionality of the TRAIL were unfavorable.

## Introduction

TNF Related Apoptosis Inducing Ligand (TRAIL), one of the immune system’s modulators, mainly is produced by various types of immune cells such as natural killer cells, T cells, dendritic cells and macrophages (1). Reportedly, TRAIL plays a considerable role in controlling immune system homeostasis, eradicating infected cells and destroying tumoral cells (2). TRAIL belongs to type II transmembrane proteins and primarily resides as Zinc ion stabilized trimer protein on the surface of immune cells (3). Active soluble trimer TRAIL is released from the surface of immune cells through cleavage of specific Cysteine protease, and induces apoptosis by interacting with type 4 and 5 death receptors (CD261 and CD262) (Cluster of differentiation) on target cells (4). Interestingly, the expression of type 4 and 5 death receptors on cancerous and infected cells is higher than their expression in normal cells; thus, TRAIL can specifically destroy its target cells with minimal side effects (5). Accordingly, numerous investigations have been conducted on the exploiting of the TRAIL as a promising therapeutic agent in autoimmune diseases and targeted cancer therapy (6). 

Due to no requirement of specific post translational modifications on the soluble TRAIL, enormous efforts have been fulfilled to produce this biopharmaceutical candidate in different expression systems. Firstly, the recombinant TRAIL was actively produced in insect cells by using baculovirus transformation approach (7). However, insect cell expression system is not ideated to be applicable methodology for industrial scale up production of TRAIL due to relatively low yield, and moderately high production cost (8). Likewise, several bacterial expression systems have plentifully been exploited to produce recombinant TRAIL (9-11). Despite possessing a remarkable yield as well as scale-up feasibility of TRAIL production, bacterial expression systems cannot be considered as a perfect production system because of refolding requirement of TRAIL from inclusion bodies, risk of endotoxins contamination, and higher purification cost (12). Similarly, enormous yeast expression systems have been utilized for TRAIL production (13,14).However, in spite of great potential of yeasts to produce biopharmaceuticals, the necessity of utilizing expensive fermentor services limits recombinant protein production in these systems (15). In the same way, mammalian cell based expression systems have been employed in TRAIL production (16,17). Although these systems are capable of producing accurately folded TRAIL, its production was seriously restricted by both the possible contamination risks with viruses/prions, and the highest cost of production and purification (12). 

Among the expression systems, plants have considerable merits in producing biopharmaceuticals (molecular farming) due to their capability to produce accurately folded proteins with lowest production cost, and no risk of pathogens contamination (18-20). As a result, regardless of having lowest expression level, plant based expression systems have been used for cost beneficial production of numerous biopharmaceuticals such as antibodies (21), vaccines (22,23), hormones (24), and cytokines (25). Correspondingly, various types of plants have been utilized for molecular farming, and among them tobacco’s species are considered as highly efficient green bioreactors due to remarkable leaf biomass, high soluble protein content and being a non-food crop (26).

Despite having promising potentials in biopharmaceuticals production, plant based expression systems have not been fully exploited for the production of the TRAIL. Therefore, the purpose of this study was to investigate the ability of *N. tabacum *cells for production of human soluble TRAIL. Accordingly, we conducted *Agrobacterium tumefaciens* mediated transformation method to transfer TRAIL encoding gene into* N. tabacum* callus cell lines. Furthermore, production yield and biological activity of the recombinant TRAIL were analyzed. 

## Experimental

*Materials*

General molecular biology reagents including RNX-Plus, Tris-Base, SDS (Sodium dodecyl sulfate), Acrylamide, Bisacrylamide and Agarose were purchased from CinnaGen, Iran. Most of the required enzymes including *pfu DNA polymerase, MMLV-rt(*Moloney Murine Leukemia Virus reverse transcriptase)*, T4 DNA Ligase, Eco*RV,* Bam*HI, *Sac*I and *Bgl*II were obtained from Fermentase*.* 35S-CaMV plasmid and pGreen II 0179 were purchased from John Innes Centre, UK. *Agrobacterium tumefaciens *LBA 4404 and *Nicotiana tabacum* cell line were kindly donated by NIGEB (National Institute of Genetic Engineering and Biotechnology) and Dr Ghanati (Tarbiat Modares University), respectively. All requirements for plant cell culture media including major and minor minerals; hormones (such as Indole acetic acid, Naphthyl acetic acid, Kinetin, Acetosyringon), vitamins and sugars (Such as Myoinositol and Sucrose) were provided from Merck, Germany. Antibiotics including Ampicillin, Kanamycin, Tetracycline, Hygromycin, Streptomycin and Cefotaxime were purchased from Sigma. Phenyl Methyl Sulfonyl Fluoride (PMSF) protease inhibitor and nitrocellulose membrane were acquired from Roche and Protino Ni-TED (Nickel-Tris(carboxymethyl)ethylene diamine) packed chromatography column was purchased from Machehery-Nagel, UK. Bio-Rad detergent compatible Kit and New England BioLab Bovine Serum Albumin were used to determine protein concentration. Recombinant TRAIL (ab168898) as standard along with TRAIL polyclonal antibody (ab2435) and anti-rabbit IgG secondary antibody (ab131365) were purchased from Abcam. A549 cell line (ATCC® CCL-185™) as a cancer model was obtained from Pasteur Institute of Iran.

*Methods*

*Preparation of Soluble Human TRAIL expression gene fragment*

In order to obtain soluble human TRAIL gene fragment, at first total RNA of human peripheral white blood cells was extracted using RNX-Plus reagent as manufacture’s instruction manual. Then, cDNA library was constructed by utilizing MMLV- reverse transcriptase and oligo-dT primers. Afterward, to obtain the extracellular region fragment of TRAIL, PCR was carried out on cDNA library by exploiting *pfu *DNA polymerase and specific (F1 and R1) TRAIL primers (Table 1 and Table 2). Subsequently, nested PCR reaction was performed by F2 and R2 primers (Table 1 and Table 2) on obtained fragment to attain encoding region of soluble human TRAIL. The final achieved fragment was confirmed by sequencing technique.

**Table 1:** Primers for PCR amplification of TRAIL

Primers	Sequences from 5' to 3'	Descriptions
F1	ACAGCCCCTGCTGGCAAGTC	Specific Forward
R1	TTAGCCAACTAAAAAGGCCCCGA	Specific Reverse
F2	GTGAGAGAAAGAGGTCCTCAGAGAG	Nested Forward
R2	GGTACCGCCAACTAAAAAGGCCCCA	Nested Reverse
F3	GGGATCC*AACAATGG*TGAGAGAAAGAGGTCCTCAGAGAG	*Bam*HI + Kozak
R3	*ATGATG*ACCTCTGCCAACTAAAAAAGCCCC	His-Tag
R4	*ATGATGATGATGATGATG*ACCTCTGCCAAC	His-Tag
R5	GGAGCTCTTA*ATGATGATGATGATGATG*ACCTCTGCCAAC	His-Tag* + Sac*I

Restriction enzymes digestion sites were represented as highlighted Bold texts.

The Kozak sequence was represented as underlined Italic text.

The C-terminal 6 His-Tag purification facilitators were represented as Italic texts.

**Table 2:** PCR programs for subsequent amplification of TRAIL.

PCR primers	Initial Denaturation	(PCR Cycles)*30	Final Extension
F1 R1	95 ^o^C (5min)	95 ^o^C (45 Sec) 61 ^o^C (40 Sec) 72 ^o^C (1 min)	72 ^o^C (10min)
F2 R2	95 ^o^C (5min)	95 ^o^C (40 Sec) 60 ^o^C (40 Sec) 72^o^C (1 min)	72 ^o^C (10min)
F3 R3	95 ^o^C (5min)	95^o^C (40 Sec) 65 ^o^C (40 Sec) 72^o^C (1 min)	72 ^o^C (10min)
F3 R4	95 ^o^C (5min)	95^o^C (40 Sec) 63 ^o^C (40 Sec) 72^o^C (1 min)	72 ^o^C (10min)
F3 R5	95 ^o^C (5min)	95^o^C (40 Sec) 66 ^o^C (40 Sec) 72 ^o^C (1 min)	72 ^o^C (10min)

*Providing expression and translation controlling regions*

**Figure 1:**
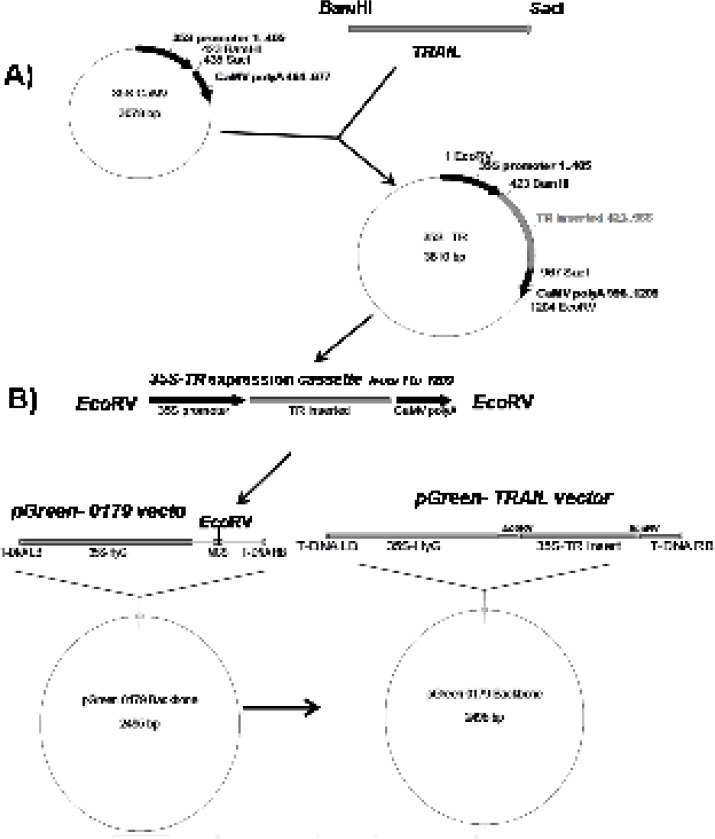
Schematic diagram of soluble human TRAIL cloning into intermediate 35S CaMV vector and pGreen-0179 plant expression vector. Panel A) represents cloning of TRAIL into *Bam*HI and *Sac*I regions of 35S-CaMV plasmid to obtain 35S promoter-TR inserted-CaMV polyA (1200 bp) fragment. Panel B) represents cloning of 35S promoter-TR inserted-CaMV polyA fragment into *Eco*RV region of the pGreen-0179 T-DNA to obtain final pGreen-TRAIL vector. LB and RB (Left border/right border) stands for left border and right border respectively.

With the aim of expressing soluble human TRAIL in plant cell system, at first, “ plant compatible 5ʹ translation initiator Kozak sequence” and “C-terminal 6 His-Tag purification facilitator” fragments were added to TRAIL encoding gene using sequential PCRs via respective (F3) and (R3, R4, R5) primers (Table 1 and Table 2). All PCR reactions were carried out by *pfu DNA polymerase* and the accuracy of the reactions was confirmed by sequencing technique. The obtained cassette was cloned between the *Bam*HI and *Sac*I regions of 35S-CaMV plasmid possessing plant specific promoter and terminator regions (Figure 1A). Finally, the designed expression construct was sub-cloned into the *Eco*RV region of pGreen II 0179 plant expression vector mainly to provide both selectable markers and *A. tumefaciens *transferable oncogenic DNA (T-DNA) regions (Figure 1B).

*Cloning into Agrobacterium tumefaciens*

*A.* *tumefaciens* LBA4404 was grown on broth Luria-Bertani medium included streptomycin (100 μg/mL) at 25 ^o^C, 200 rpm for 48 hours. The final obtained TRAIL expressing pGreen vector and replication facilitator pSoup helper plasmid (27) were co-transformed to *A. tumefaciens* LBA 4404 using previously described freeze-thaw method (28). Transformed bacteria were selected on Kanamycin (50 μg/mL), Tetracycline (2 μg/mL) and Streptomycin (100 μg/mL) selection medium. To induce the virulent genes of *Agrobacterium*, the transformed bacteria were grown on broth selection medium containing 100 μM Acetosyringon for at least 2 hours.

*Transformation and selection of Nicotiana tabacum cells*

*N. tabacum* callus cells were grown at 25 ^o^C in the darkness condition on solid LS medium (Linsmaier & Skoog Medium) (29) supplemented with Indole acetic acid 3 μg/mL, Naphthyl acetic acid 3 μg/mL, Kinetin 100 ng/mL, Myoinositol 100 μg/mL and Sucrose 30 mg/mL Since Zn^2+^ plays important role in proper assembly of the TRAIL, two fold of Zn^2+^ ion regular concentration was used for LS medium preparation. Transformation of *N. tabacum* cells was performed by co-cultivation technique (30) with some modifications. At first, 10 mL of freshly activated transformed *A. tumefaciens* (OD: 1, 600 nm) (Optical Density) was centrifuged (3000 rpm, 5 min, 25 ^o^C) and the medium was replenished by 5 mL modified LS medium containing 100 μM Acetosyringon. Afterward, one gram of freshly grown *N. tabacum* cells was added to the bacterial culture and was incubated at 100 rpm about 2 h at 25 ^o^C. The incubated cells were centrifuged (1000 rpm, 10 min, 25 ^o^C) and the cells pellet was placed on the sterile filter paper for 5 min to wipe excess water. Inoculated plant cells were subsequently transferred to the solid modified LS medium containing 100 μM Acetosyringon and were kept for two days at 25 ^o^C in the darkness condition. Remained *Agrobacterium* cells were eradicated by means of sub-culturing on medium containing Cefotaxime (200 μg/mL). Finally, transformed *N. tabacum* cells were screened through frequent sub-culturing on selection medium containing Hygromycin (30 μg/mL) and Cefotaxime (200 μg/mL) every two weeks. Selected *N. Tabacum *cells in the week 8^th^ were used for the rest of the experiments.

*Total protein extraction and TRAIL purification*

Two grams of transformed *N. tabacum *cells were suspended into 5 mL ice cold non-reducing extraction buffer (PBS pH: 7.4, Glycerol 10%, PMSF 1 mM) (Phosphate Buffered Saline) and were homogenized using Heidolph silent crusher (20000 rpm, 45 seconds). The mixture was placed on ice for 10 min and was centrifuged twice (12000 rpm, 15 min, 4^ o^C). The supernatant was filtrated through 0.45 μm filter to remove remained solid materials. Meanwhile, total protein concentration was measured using Bio-Rad DC kit (based on Lowry method), where bovine serum albumin was utilized as a standard. To purify recombinant TRAIL, total protein extract of transformed *N. tabacum *cells was passed through pre-equilibrated Ni-TED pre-packed column and the purity of the TRAIL was confirmed by SDS-PAGE.

*Western blot analysis*

A total of 1 mg of acetone precipitated total protein extract from both transformed and untransformed plant cells was denatured by boiling in 100 μL non-reducing loading buffer (0.225 M Tris Cl, pH 6.8, 50% glycerol, 5% SDS) for 5 min. Subsequently, 200 μg of the protein samples along with pre-stained protein marker (Biobasic, RM0011, Korea) were separated on a 15% SDS-PAGE. Afterward, the separated proteins were transferred to a nitrocellulose membrane by semi-dry technique (Towbin buffer, 200 mA, 2 h, Apelex PS 304). The proper transfer of proteins to the membrane was indicated by the color of the pre-stained protein marker which remains the same even after blotting. After attaching the proteins to the nitrocellulose membrane by ultra violet cross linking method (120 m Joules, 1min, UviTech), the membrane was blocked by incubating overnight in PBS buffer (pH 7.4) containing 5% (w/v) skimmed milk at 4 ^o^C. Following washing three times in Tris-Buffered Saline (TBS: 20 mM Tris-HCl, 140 mM NaCl, pH 7.5) at 200 rpm for 10 min, the membrane was incubated for 2 h (80 rpm) with a rabbit polyclonal antibody of TRAIL (1:1000; Abcam, ab2435) in TBS-T buffer (TBS plus 0.1% Tween-20, v/v) containing 1% (w/v) skimmed milk. Afterward, the membrane was subjected to three times washing procedures with TBS and TBS-T buffers (10 min, 200 rpm). Subsequently, the membrane was incubated for 1 h (80 rpm) with alkaline phosphatase conjugated anti-rabbit IgG secondary antibody (1: 5000; Abcam, ab131365) in TBS-T buffer, containing 1% (w/v) skimmed milk. After several washing steps (5 min, 200 rpm) with TBS and TBS-T buffers, the membrane was incubated with Bromo Chloro Indolyl Phosphate (BCIP) and Nitro Blue Tetrazolium (NBT) reagents in alkaline phosphatase buffer (100 mM NaCl, 5 mM MgCl_2_, pH 9.5) for color development. Finally, the color development was stopped by washing the membrane with distilled water.

*Semi-quantitative western blot analysis*

A total of 200 μg from crude protein extract of recombinant *N. tabacum* samples and 1 μg of recombinant human TRAIL (ab168898) as a standard were exploited to SDS-PAGE and semi quantitative western blot analyses. The intensity of developed bands was analyzed using ImageJ software and data were utilized to calculate approximate amount of TRAIL production in transformants.

*Functional assay*

The anti-proliferation activity of TRAIL on A549 cells line was determined using MTT assay as previously described (31). Briefly, A549 cells were cultured at a seeding density of 4.0 × 10^4^/cm^2^ in 96-well micro plate, each containing 200 μL of the growth medium (RPMI-1640). The cells were grown in the humidified incubator at 37 ^o^C with 5% CO_2_, until they reach about 40% confluence. Total protein extracts of both transformed and untransformed *N. tabacum *cells were concentrated with Centricon filter concentrators (10 kDa cutoff). A total of 200 μg of concentrated protein extracts, as well as 1 μg His tag purified TRAIL were added to each well, following by further incubating for 48 h at cultivation condition. Correspondingly, 100 ng recombinant human TRAIL (ab168898) and 50 μL extraction buffer were used as positive and negative controls, respectively. After incubation period, the medium was replaced with 200 μL fresh medium containing extra 50 μL of MTT solution (2 mg/mL in PBS), and the cells were incubated for an additional 4 h at 37 ^o^C. Afterward, the medium was completely removed and 200 μL DMSO (Dimethyl sulfoxide) in addition to 25 μL Sorenson buffer (0.1 M glycine, 0.1 M NaCl, pH 10.5) were added to each well. The absorbance of each well was measured by employing a BioTek Synergy 3 micro plate reader at 570 nm. Obtained data were demonstrated as Mean ± SD. Results were implemented by Excel (version 2007) and SPSS (version 16). Statistical analyses between mean values were performed using one-way analysis of variance (ANOVA) and post test of least significance difference (LSD). p-value less than 0.05 was considered as significant difference.

## Result and Discussion

*Obtaining TRAIL encoding cassette*

**Figure 2:**
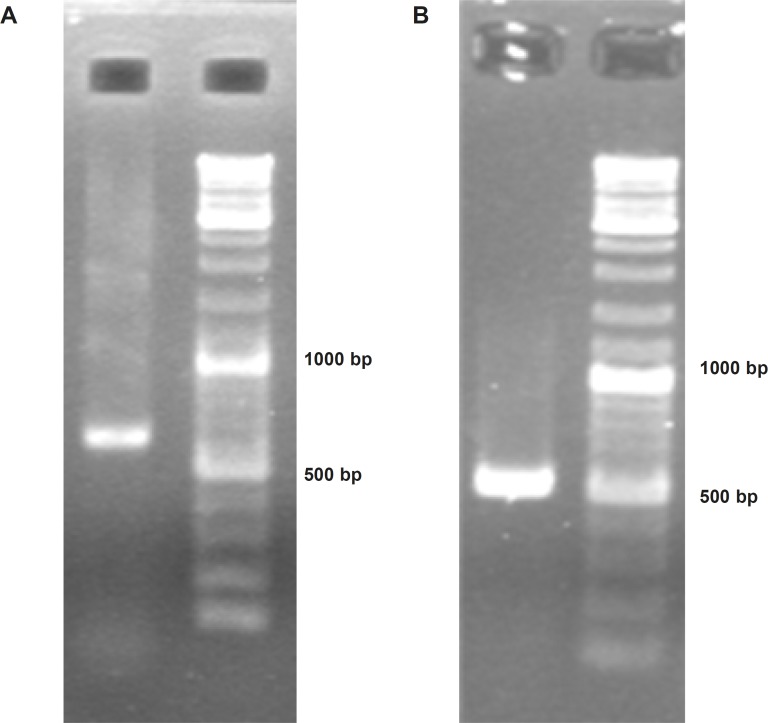
Confirmation of soluble human TRAIL PCR products using electrophoresis on 2% Agarose gel. Panel (A) represents obtaining “a 620 bp fragment” from extracellular domain of TRAIL through specific RT-PCR. Panel (B) represents obtaining a 504 bp band encoding soluble human TRAIL through nested PCR.

To construct Soluble Human TNF Related Apoptosis Inducing Ligand (ShTRAIL) encoding cassette, at first RT-PCR was performed on isolated total RNA of human peripheral white blood cells, and specifically about 620 bp fragment of TRAIL’s extracellular region was obtained (Figure 2A). Subsequently, the desired ShTRAIL gene (504 bp) encoding region was acquired by nested PCR (Figure 2B). Furthermore, the translation controlling regions were also adjoined using sequential PCRs; and the accuracy of final ShTRAIL encoding cassette (551 bp) was confirmed by sequencing technique. 

*Constructing TRAIL encoding expression vector*

**Figure 3:**
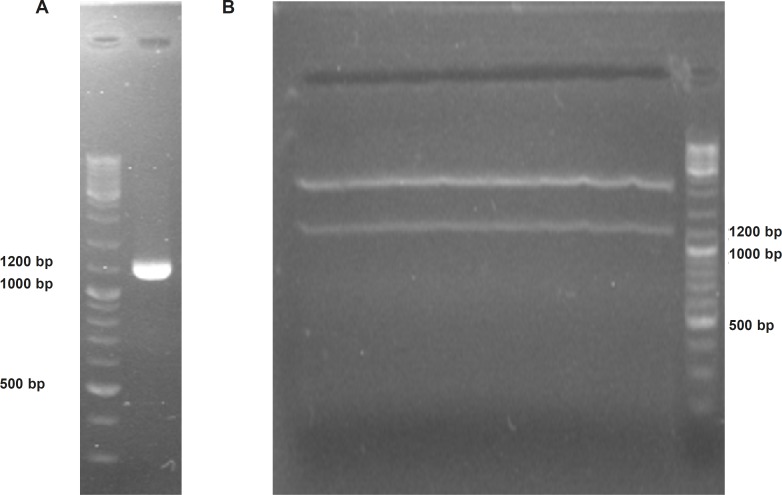
Confirmation of soluble human TRAIL cloning into 35S-TR plasmid using electrophoresis on 2% Agarose gel. Panel (A) represents obtaining a 1209 bp “35S promoter-TR inserted-CaMV polyA fragment” via PCR. Panel (B) demonstrate separation of cloned 1200 bp TRAIL expressional region from 35S-TR plasmid through *Eco*RV digestion.

**Figure 4:**
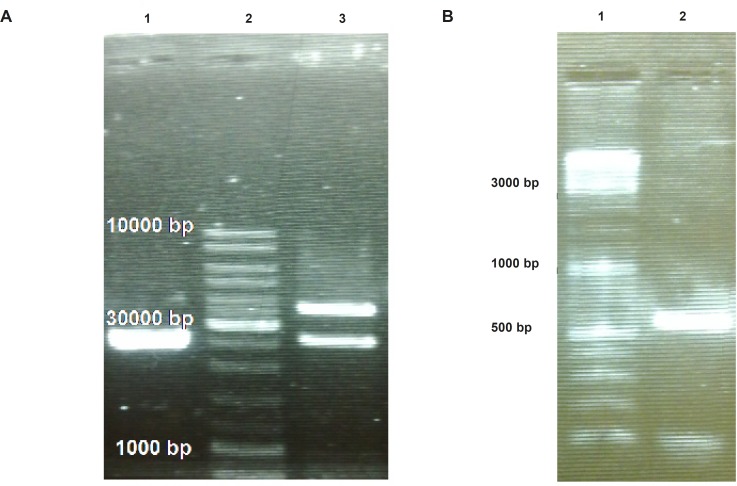
Confirmation of “35S promoter-TR inserted-CaMV polyA fragment” cloning into pGreen-0179 plasmid using electrophoresis on 2% Agarose gel. Panel (A) represents separation of the pGreen-0179 backbone (2495 bp) from the T-DNA region of the empty (2648 bp) and TRAIL contained (3860 bp) pGreen-0179 vector via *Bgl*II enzyme digestion: The empty pGreen-0179 (lane 1), DNA Ladder (lane 2), pGreen-TRAIL (lane 3). Panel (B) represents TRAIL specific PCR using F3R5 primers on TRAIL cloned pGreen-0179 vectors (lane 2) and DNA ladder (lane 1).

To provide plant compatible promoter and terminator regions, the ShTRAIL encoding cassette was sub-cloned into intermediate 35S-CaMV plasmid between *Bam*HI and *Sac*I restriction enzymes regions, named 35S-TR (Figure 1A). After inserting the ShTRAIL encoding cassette (551 bp) into the 35S-CaMV plasmid expression cassette region (677 bp), the total size of “35S promoter-TR inserted-CaMV polyA” fragment was raised to about 1200 bp (35S-TR expression cassette) and the result was verified by both PCR and digestion methods (Figure 3). Then, the 35S-TR expression cassette was sub-cloned into *Eco*RV region of pGreen 0179 binary vector to produce final plant TRAIL expression vector, pGreen-TR plasmid (Figure 1B). The insertion of the 35S-TR expression cassette was confirmed through *Bgl*II enzymatic digestion (Figure 4a) and TRAIL specific PCR (Figure 4b) using F3R5 primers (Table 1 and Table 2). Finally, the pGreen-TR plasmid was used to transform *N. tabacum *callus cells using *Agrobacterium *mediated system. 

*Expression analysis of TRAIL in transformed N. tabacum callus cells*

**Figure 5:**
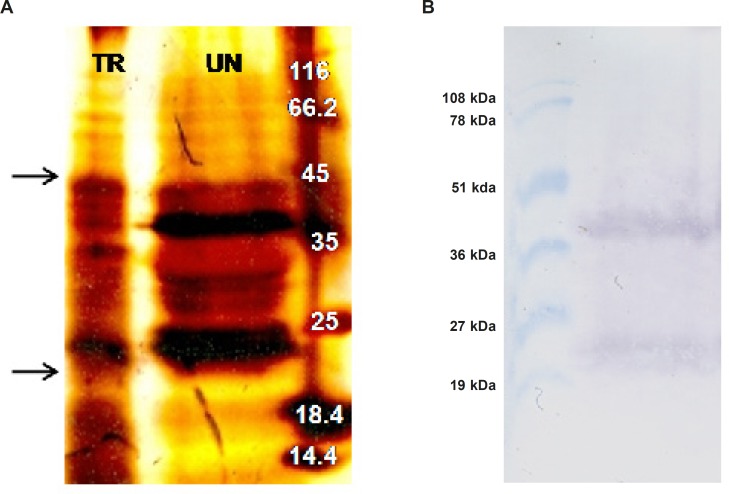
SDS-PAGE and western blot analyses of ShTRAIL obtained from transformed *N. tabacum* callus cells (A) silver staining of the transformed (TR) and untransformed (UN) *N. tabacum* total protein extract on 15% PAGE. The arrows indicate bands of monomer and dimer forms of TRAIL (B) Obtaining TRAIL specific monomer (22 kDa) and dimer (44 kDa) bands through western blot analysis of selected recombinant *N. tabacum *callus cells.

In an attempt to investigate expression of ShTRAIL, total protein extract of transformed and untransformed *N. tabacum *cells were analyzed by SDS-PAGE (Figure 5A) and western blot techniques (Figure 5B) in non-reducing condition. 

Considering molecular weight of the designed recombinant ShTRAIL’s monomer (22 kDa), the presence of both 22 and 44 kDa bands in non-reducing western blot suggested successful production of recombinant ShTRAIL by *N. tabacum* cells (Figure 5A). Correspondingly, our finding is compatible with previously reported results regarding the presence of just monomer and dimer TRAIL in non-reducing SDS-PAGE and western blot analyses (32). However, as shown in Figure 5B, the lack of counterpart intense bands in SDS-PAGE analysis perhaps related to the low level of recombinant protein production in plant based expression systems (18). 

*Estimation of TRAIL expression level in N. tabacum callus cells*

**Figure 6:**
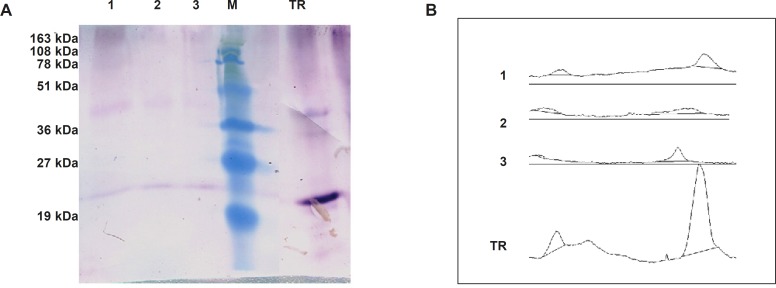
Estimation of TRAIL production by Semi-quantitative western blot analysis. Panel (A) represents western blot analysis of 200 μg total protein extract of transformed *N. tabacum* cells (1,2,3) beside 1 μg of recombinant standard TRAIL (TR). Panel (B) represent corresponding area of developed bands through ImageJ software analysis.

To speculate TRAIL’s expression level using semi-quantitative western blot, densitometry analysis of developed bands roughly suggested that up to 2.5 μg of TRAIL could be obtained from 200 μg loaded total protein (Figure 6). This expression level was corresponded to 13.5 μg TRAIL in one gram fresh weight of *N. tabacum *callus cells (Table 3). This amount of expression is slightly lower than previously described recombinant protein production level in *N. tabacum *callus cells; 30 μg/g monoclonal antibody (33); 50 μg/g human tissue transglutaminase (34); and, 20 μg/g human serum albumin (35). However, our results do not support Wang *et al*.’s reports on the unsuccessful production and accumulation of the TRAIL in transplastomic *Tobacco* (36). Probably the prime causes of this discrepancy are related to the differences of the studies regarding the systems of expression and the varieties of the hosts. Correspondingly, several other investigations underline the importance of optimizing expression cassette and host variety on the recombinant protein yield (37,38).

**Table 3:** Relative expression level of ShTRAIL in transformed *N. tabacum.*

**Different selected Callus cells**	**Total soluble protein in Crude extract (mg/mL) based on Lowry method**	**TRAIL in crude extract (μg/mL) semi quantitative western Blot**	**TRAIL % in Soluble protein Extract**	**TRAIL (μg) in 1 g of ** ***N. Tabacum***
1	1.2 ± 0.1	2.5	0.21	13.5
2	1.0 ± 0.1	1.6	0.16	10.5
3	0.9 ± 0.2	1.3	0.14	9.2
4	1.2 ± 0.03	1.1	0.10	7.9

*Purification of recombinant TRAIL*

**Figure 7:**
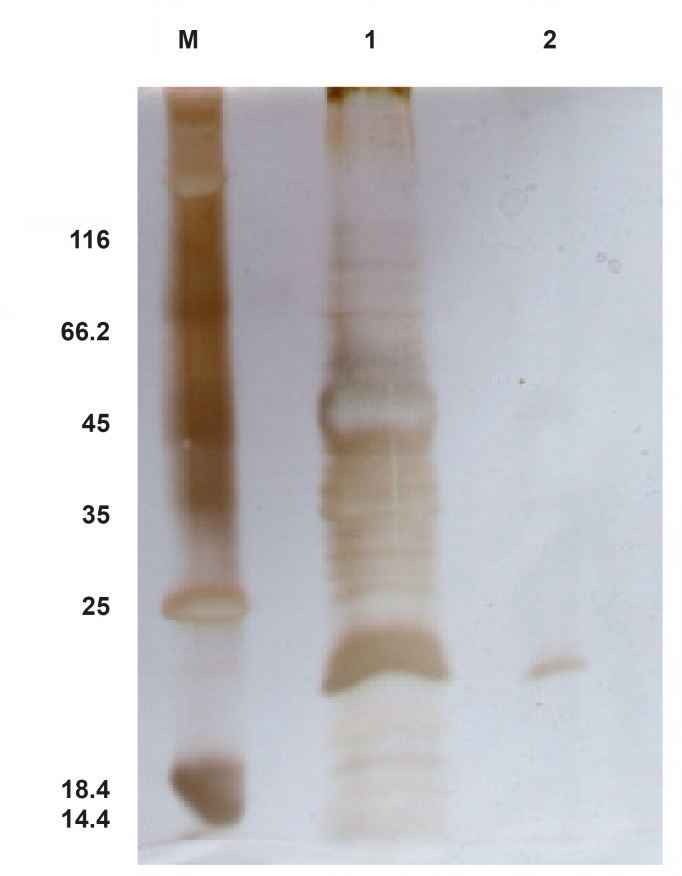
Confirmation of TRAIL Purification through silver staining method. Unstained Fermentase protein ladder #SM0431 (lane M), crude protein extract (lane 1), purified monomer and dimer forms of TRAIL (lane 2) are represented.

In an attempt to purify ShTRAIL from the crude extract, His-Tag affinity chromatography procedure on Ni-TED column was performed. Non-reducing SDS-PAGE analysis of eluted fractions revealed that likely about 80% pure ShTRAIL can be obtained in this procedure (Figure 7). However, the majority of the obtained ShTRAIL proteins were in their monomer and oxidized dimer states in the purification condition (pH: 8, imidazole 250 mM). Moreover, due to low expression level of the His-tagged ShTRAIL, the majority of the Ni-TED column binding sites were occupied with undesirable proteins which were eluted along with ShTRAIL during the purification process (Figure 7).

*Functional assay of recombinant TRAIL*

To evaluate biological activity of *N. tabacum *produced ShTRAIL, MTT assay on A549 cell line was performed. Undesirably, there was not significant recurrent antiproliferative effect of neither purified TRAIL nor recombinant crude extract protein on A549 cells in comparison with their untransformed counterparts after 48 h (*p*>0.05) (Data were not shown). This limited activity probably attributed to both the low expression level and absence of successful trimeric assembly of TRAIL, which are critical factors for its biological function (4). Similarly, Kim and colleagues report that both secretable and non- secretable forms of native TRAIL, produced in the HEK 293 (Human Embryonic Kidney 293) mammalian cells, do not possess significant apoptotic activity due to low expression level and lack of successful trimerization of the TRAIL (39). On the contrary, while prokaryotic expression systems produce TRAIL mostly as inclusion bodies, the refolded and purified TRAIL retains its biological activity and produces trimeric form possibly due to higher production level of prokaryotic systems (9,32,40).

Furthermore, as non-reducing SDS-PAGE and western blot analyses indicated (Figure 5B and Figure 6), despite providing Zn^2+^ during ShTRAIL production and even concentrating ShTRAIL in purification procedure, which both are in favor of TRAIL trimerization, the majority of ShTRAIL are still in monomer and dimer forms. This lack of successful assembly of *N. tabacum* produced TRAIL contrast with previous reports on proper assembly of other multi-subunit proteins such as human homotrimeric collagen (41,42) and several functional antibodies (21,43) in plant based expression systems. The prime reason for the discrepancy may be due to the different types of stabilizing forces for these self-assembled molecules. While collagen and antibody molecules were stabilized by various hydrogen and covalent bonds, trimer TRAILs were assembled via weak electrostatic interactions between Cysteine 230 residues and Zn^2+^ (3,44). Therefore, probably dissociation of fragile electrostatic bonds during extraction procedure of ShTRAIL from *N. tabacum *cells leads to disassembling of trimer TRAILs. Accordingly, as stated previously, once trimer TRAILs is disassembled, the buried Cysteine 230 residues are exposed to oxidizing environment and low active disulfide-linked dimer TRAIL is generated (44). Consequently, as disulfide-linked dimer TRAIL cannot participate in Zn^2+^ stabilized trimer TRAIL assembly, employment of higher concentration of the TRAIL cannot guarantee the proper trimer assembly of the TRAIL (44). Besides, the ED50 (Median effective dose) of the TRAIL in the A549 cell line is about 200 ng/mL (45,46). Despite employing 5 μg/mL of purified recombinant TRAIL (25 times higher concentration than ED50), we did not recognize any significant TRAIL antiproliferative activity on A549 cell line. Consequently, further optimization of recombinant TRAIL purification and activity assay conduction will be required for better biological activity evaluation of *N. tabacum * produced ShTRAIL.

## Conclusion

Molecular Farming of ShTRAIL in *N. tabacum *using *Agrobacterium tumefaciens* LBA 4404 suggested that while production of self-assembled protein is technically feasible by this method, functional purification of such molecules endure much more efforts. Despite retrieving about 15 μg recombinant TRAIL per one gram fresh weight cell, *N. tabacum* extracted TRAIL mostly was in inactive dimer form. Therefore further work on extraction and purification optimization seems to be mandatory.
